# Globular and disordered—the non-identical twins in protein-protein interactions

**DOI:** 10.3389/fmolb.2015.00040

**Published:** 2015-07-09

**Authors:** Kaare Teilum, Johan G. Olsen, Birthe B. Kragelund

**Affiliations:** Structural Biology and NMR Laboratory, Department of Biology, University of CopenhagenCopenhagen, Denmark

**Keywords:** ITC, IDP, intrinsically disordered, entropy, enthalpy, stability

## Abstract

In biology proteins from different structural classes interact across and within classes in ways that are optimized to achieve balanced functional outputs. The interactions between intrinsically disordered proteins (IDPs) and other proteins rely on changes in flexibility and this is seen as a strong determinant for their function. This has fostered the notion that IDP's bind with low affinity but high specificity. Here we have analyzed available detailed thermodynamic data for protein-protein interactions to put to the test if the thermodynamic profiles of IDP interactions differ from those of other protein-protein interactions. We find that ordered proteins and the disordered ones act as non-identical twins operating by similar principles but where the disordered proteins complexes are on average less stable by 2.5 kcal mol^−1^.

## Introduction

Proteins function though the action and communication with other molecules and the intricate interplay among residues within every binding site results in diagnostic thermodynamic profiles implicit to the particular molecular pair. In protein-protein interaction the majority of the binding energy comes from a few critical hot-spot interactions (Clackson and Wells, 1995), but the binding energy also depends on other factors such as interface size, residue composition, flexibility of the interacting partners as well as on environmental cues. The discovery of a large fraction of the proteome being intrinsically disordered (ID) means that a substantial fraction of protein-protein interactions involves proteins or parts of proteins, which do not adopt a well-defined three-dimensional structure in the unbound state. These proteins, or regions in proteins, originate from the class of *intrinsically disordered proteins* (IDPs) (Dunker et al., 2000; Tompa, 2002; Nilsson et al., 2011). They are central to a plethora of key biological processes, are multi-specific and possess a versatile interaction potential placing many of them centrally in cellular hubs (Han et al., 2004). The prevailing notion is that IDPs are able to bind with high specificity, but low affinity, although recent kinetic studies suggest that this concept may not be straightforward (Dogan et al., 2014; Iesmantavicius et al., 2014; Krieger et al., 2014). IDPs contain very few hydrophobic residues (Dunker et al., 2001), which suggests that their interaction energies may be comparatively low, substantiated by the entropy loss of ordered complex formation from a disordered peptide chain. Specificity, on the other hand, arises when the polypeptide chain adopts the correct conformation in which the distribution of side chains match electrostatic and hydrogen bonding donors and acceptors as well as hydrophobic patches on the target. This paradigm of lower affinity of IDPs compared to globular proteins has been suggested but never challenged by a large-scale thermodynamic assessment, which is the aim of the present paper.

## Results and discussion

**Figure 1:**
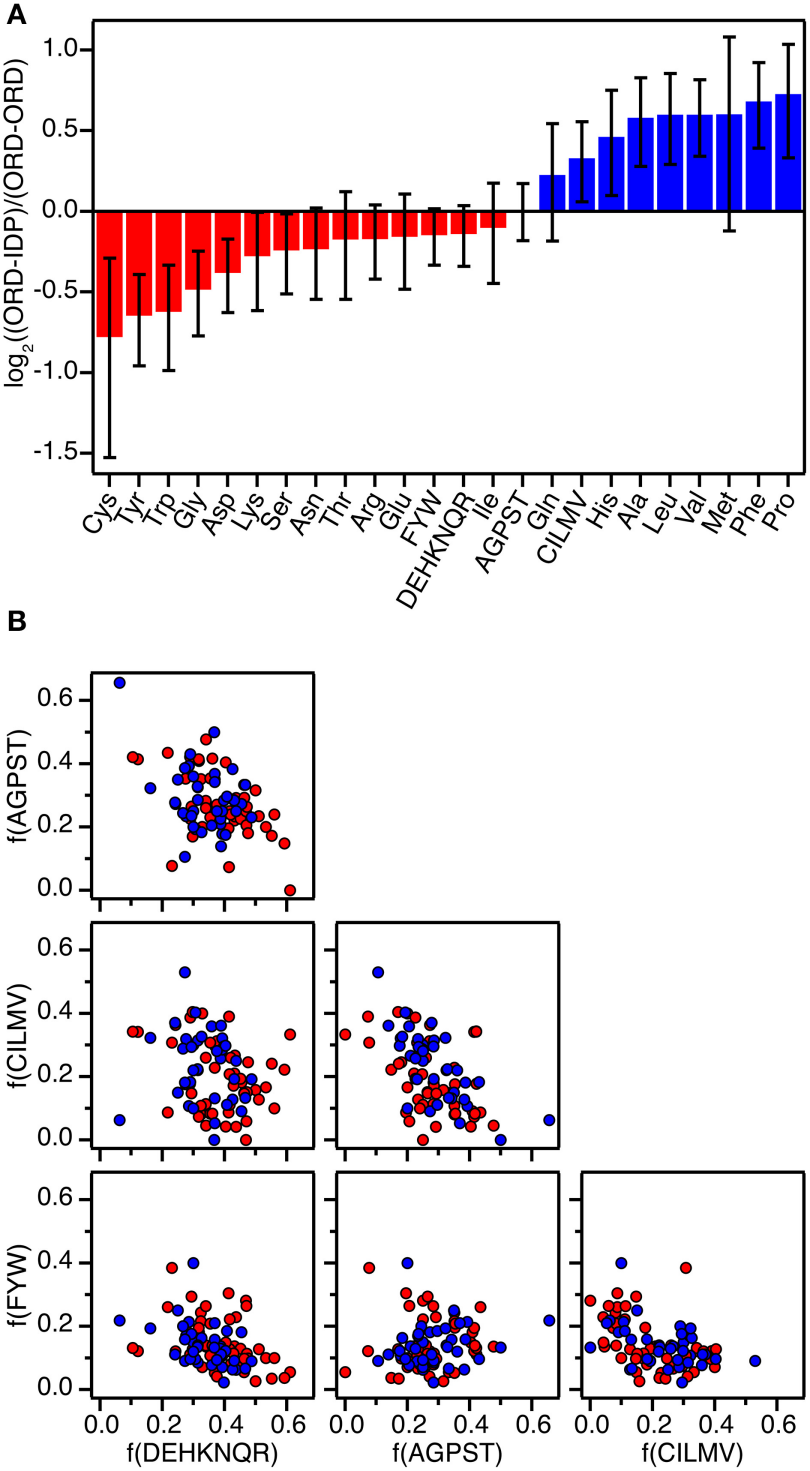
**Amino acid composition of protein-protein interfaces extracted from 87 high-resolution structures of protein-protein complexes. (A)** Fractional overrepresentation of each amino acid residue type and of the four amino acid residue classes (FWY, CILMV, AGPST, and DEHKNQR) in ORD-IDP complexes relative to ORD-ORD complexes. log_2_ of the ratios are plotted with positive values indicating overrepresentation in ORD-IDP complexes. **(B)** Correlation plots of the fractions of the four amino acid residue classes (FWY, CILMV, AGPST, and DEHKNQR) in protein-protein interfaces. Each point represents a protein-protein complex and is colored either red (ORD-ORD) or blue (ORD-IDP).

Based on previous collections of data (Stites, 1997; Huang and Liu, 2013) and including several additional data from the literature found by searching PubMed for “ITC protein-protein interactions,” “ITC intrinsically disordered protein,” “thermodynamics protein-protein interactions,” and “thermodynamics intrinsically disordered protein,” we have compiled thermodynamic parameters from close to 200 different protein-protein interaction studies (Supplementary Table 1). The data were standardized to 298 K assuming that ΔC_p_ = 0, as ΔC_p_ has only been estimated for very few of the complexes. We have estimated that the error introduced in ΔG^0^ is less than 0.2 kcal mol^−1^ in the most extreme cases where the data were measured at 281 K. For most cases where there is less than 5 K difference the error is less than 0.05 kcal mol^−1^. We subsequently compared and correlated the parameters for interactions that involve only globular proteins (91 complexes), to the parameters for interactions, where one partner is an IDP (106 complexes). To avoid over-representing a single protein-protein complex we exclusively compared wild-type proteins so that protein specific irregularities will be averaged out. In the cases where a structure of the complex has been determined, we have calculated the interaction surface area using PISA (Krissinel and Henrick, 2007) (Supplementary Table 1), and determined the amino acid composition of the interface using NCONT from the CCP4i suite (Winn et al., 2011). The amino acids were divided into four classes for analysis (FWY, CILMV, AGPST, and DEHKNQR) based on the BLOSSUM50 substitution matrix as defined by Weathers et al. (2004). The interfaces of the all ordered (ORD-ORD) complexes and the ordered-IDP (ORD-IDP) complexes were then compared in this context (Figure 1).

Complexes from the two groups were almost equally represented (ORD-ORD: 52 structures average interface size of 886 ± 46 Å^2^; ORD-IDP: 41 structures, average interface size of 905 ± 80 Å^2^) in the protein data bank (Berman et al., 2000). The sizes of the binding interface areas in the two groups of proteins were not significantly different (*t*-test, *P* > 0.05), (Supplementary Table 1). This is perhaps not unexpected, although one might have anticipated the IDP-complexes to have—on average—smaller interfaces, as many of their interactions are mediated by small linear motifs (SLiMs) (Dinkel et al., 2014), and short molecular recognition motifs (MoRFs) (Mohan et al., 2006). These motifs are typically peptide regions that fold into regular secondary structure on binding. Thus, one conclusion is that in the globular complexes analyzed here, there are equally many small interfaces, matching those of SLiMs and MoRFs of IDPs.

The second result of the structural analysis is that the intermolecular interactions, as reflected in the distribution of the four groups of amino acids, is the same (Figure 1). This observation is perhaps more surprising since the amino acid composition of IDPs is very distinct and different from that of globular proteins (Weathers et al., 2004; Uversky et al., 2005; Han et al., 2006; Hansen et al., 2006) with the low content of hydrophobic residues as the underlying reason for IDPs not forming globular structures. However, the amino acid composition on the surface of globular proteins seems to resemble that of IDPs more than the overall composition (Fukuchi and Nishikawa, 2001; Tompa, 2002; Levy, 2010). Moreover, it differs significantly from the composition of interfaces in obligate oligomers that are typically much more hydrophobic (Janin et al., 2008). In a previous study the residue composition of extended binding surfaces of IDPs bound to an ordered partner was investigated (Wong et al., 2013). Compared to interfaces between two ordered proteins, the IDPs in complex with an ordered partner had in that work an overrepresentation of hydrophobic residues as leucine and isoleucine in the core of the interface, and the ordered binding partner had an increased number of charged residues. Thus, this apparent counter balance is in full accordance with the overall sum of the interface we report here. A decomposition of the distribution into individual residues within the current set supports previous findings, although the effect is small (the largest difference is for Cys which is 41% less abundant in the ORD-IDP complexes) (Figure 1A). Therefore, if specificity is embedded in interactions between charged and polar side-chains in the interface (Eaton et al., 1995; Wong et al., 2013), we find no indication to suggest that the IDPs bind to globular proteins with higher specificity than globular proteins do.

**Figure 2:**
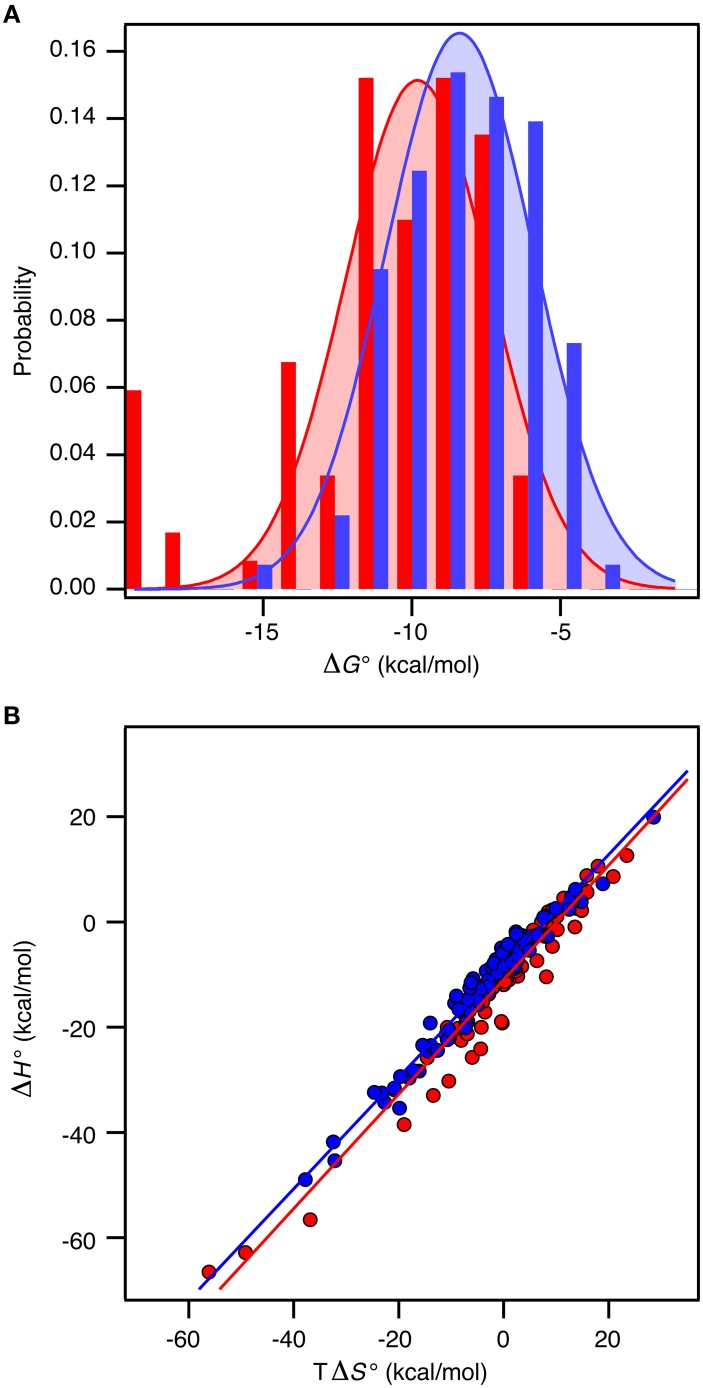
**Thermodynamics of 196 protein-protein complexes. (A)** Histogram of the binding free energy, ΔG°, for complexes between two ordered proteins (red) and one ordered and one disordered protein (blue). Both distributions were fit to a Gaussian distribution (solid lines). **(B)** Plot of ΔH° versus TΔS° for the same protein–protein complexes with the same color code as in **(A)**. The solid lines represent the best linear fits to the data.

Recall the basic thermodynamic relation, ΔG^0^ = ΔH^0^ − TΔS^0^ in which the entropy-enthalpy compensation infers that ΔH^0^ and TΔS^0^ are highly correlated (Brady and Sharp, 1997; Williams et al., 2004; Teilum et al., 2009). Thus, ΔG^0^ for the complexes in the selected sets covers a narrow range from −19.8 kcal mol^−1^ to −4.2 kcal mol^−1^ (corresponding to *K*_d_ from 3 fM to 830 μM) compared to ΔH^0^ and TΔS^0^ that are found in the ranges from −66.7 to 19.9 kcal mol^−1^ and from −56.1 to 28.5 kcal mol^−1^, respectively. The analysis of the thermodynamic parameters shows that the enthalpy (ΔH°) and the entropy (ΔS°) for binding are *not* significantly different between the two groups of proteins (*t*-test, *P* > 0.1). However, the average entropic contribution (−TΔS°) to the binding free energy for interactions between two ordered proteins is 2.5 ± 1.6 kcal mol^−1^ smaller (more stabilizing) than for interactions between an ordered and a disordered protein. Within both groups there is a linear correlation between TΔS° and ΔH° (ORD-ORD: slope = 1.09 ± 0.03, *r* = 0.97; ORD-IDP: slope = 1.06 ± 0.02, *r* = 0.98), which demonstrates a similar entropy-enthalpy compensation (Figure 2A). Thus, the same underlying thermodynamic principles are true for both groups.

In contrast to ΔS° and ΔH°, there is a significant difference in ΔG° between the groups (*t*-test, *P* < 0.0001). For the ORD-ORD complexes <ΔG°> = −11.1 ± 0.4 kcal mol^−1^, and for the ORD-IDP complexes <ΔG°> = −8.5 ± 0.2 kcal mol^−1^. The difference in <ΔG°> is 2.5 ± 0.4 kcal mol^−1^, which is primarily accounted for by the difference in TΔS° (*vide supra*). This number is close to the 2.6 kcal mol^−1^ recently published from a much smaller dataset based on mutation studies (Huang and Liu, 2013). Note that the distribution of ΔG° among the complexes for which a structure is available is similar to the distribution in the full dataset, and that this is true for the difference in <ΔG°> too. As we see no differences in the sizes of the binding interfaces or the amount of hydrophobic residues in the interfaces, and since the disordered proteins in the ORD-IDP complexes rarely form extended hydrophobic cores in their folded conformations, the hydrophobic surface area buried in ligand binding process must be similar in the two classes of protein complexes. Consequently, the difference in TΔS° is unlikely to arise from significant differences in the desolvation entropy contribution. This conclusion is in contrast to a computational study of complexes involving extended IDPs, which were selected based on a radius-of-gyration criterion of the three-dimensional structure of the complex (Wong et al., 2013). However, in that work the energetic terms were not decomposed into enthalpic and entropic contributions. Nevertheless, the experimental data for the large group of complexes that we have compiled suggest to us that the less favorable entropic contribution for the ORD-IDP complexes primarily originates from loss in conformational entropy. Indeed, it agrees with the mechanistic difference between binding an ordered and a disordered ligand. The disordered polypeptide has to fold to form the final complex, which inherently will be associated with a relative large loss in conformational entropy. It is important to note that it is not possible to conclude from equilibrium ITC data *when* the folding of the ligand occurs during the binding process. It is highly likely that for some of the protein complexes the IDP folds and then binds in a conformer selection process while for others the IDP folds upon binding in an induced fit process. The difference in <ΔG°> may still, however, be explained by the required folding of the disordered ligand in the ORD-IDP complexes.

We next analyzed the distribution of the ΔG°-values for the ORD-ORD and ORD-IDP complexes (Figure 2B). Interestingly, the most stable complexes (ΔG° <−15 kcal mol^−1^) are exclusively formed between two ordered proteins, and the least stable complexes (ΔG° ~ −5 kcal mol^−1^) are exclusively formed between an ordered and a disordered protein. Among the most stable complexes we find several enzyme: inhibitor complexes, such as the bacterial DNAses in complex with bacterial immunity proteins (Keeble et al., 2006). These DNAses form both very stable cognate complexes and less stable non-cognate complexes with immunity proteins. All these complexes are formed with similar on-rates in the order of 10^7^ M^−1^ s^−1^, and the stronger binding is achieved by slower off rates (Keeble and Kleanthous, 2005; Keeble et al., 2006). Another strong binding complex is that of barnase and barstar which has become a classical example where the electrostatic surfaces of the proteins have evolved to enhance the on-rate (*k*_ass_ = 10^8^ − 10^9^ M^−1^ s^−1^) (Schreiber and Fersht, 1996). Similar fast on-rates are reported for IDPs (Arai et al., 2012; Rogers et al., 2013; Dogan et al., 2014) and similar on-rate dependence on electrostatics has been noted (Rogers et al., 2013). Consequently, the main difference between ORD-IDP and ORD-ORD complexes seems not to reside in on-rate differences, but may therefore reside in off-rates, noted earlier in comparative kinetic studies (Huang and Liu, 2009; Shammas et al., 2012; Dogan et al., 2014). It is still possible that the electrostatic influence from a globular binding partner will cause an induction of a binding-competent conformation within the ensemble distribution of the IDP. A result of this is that it can potentially influence the on-rate and subsequently the binding energy. Alternatively, the binding-competent conformation of the IDP may be required to guide it into the electrostatic field of the globular partner. We do not currently have any data to elaborate further on these scenarios.

One of the hall-marks of IDPs is their ability to interact with many different proteins, for instance in cellular hubs (Oldfield et al., 2008; Cumberworth et al., 2013). Based on computational analyses of structures from a large set of both ordered and disordered hub-complexes from yeast, it was suggested that the binding energies become weaker as the number of interacting proteins increases (Carbonell et al., 2009). Thus proteins with only one binding partner bound with higher affinity than promiscuous proteins with more than on binding partner. This difference may possibly be caused by a broader distribution of hot spots in the promiscuous proteins (Carbonell et al., 2009). It is possible that the difference in average binding affinity <ΔG°> observed in the current set is related to an increased number of interacting partners. We have no data on the number of alternative binding partners for complexes in our analysis. Still, it is interesting to note that the difference in binding energy between specific-to-specific complexes and specific-to-promiscuous was 0.08 ± 0.01 kcal mol^−1^ residue^−1^ (Carbonell et al., 2009), which with an average of 47 residues in the interfaces provided in our data set, amounts to 3.8 ± 0.5 kcal mol^−1^, close to the average difference of 2.5 ± 0.4 kcal mol^−1^ that we found between the ORD-ORD and the ORD-IDP complexes.

One alternative explanation for the lower average stability of IDP-ORD complexes may be purely technical and unrelated to any *de facto* differences between globular proteins and IDPs. The vast majority of the experimental studies in our set are conducted on recombinant proteins, typically expressed in *Escherichia coli*. Since phosphorylations and other post translational modifications are widespread in IDPs and is a way of regulating their activity (Iakoucheva et al., 2004) the 2.5 kcal mol^−1^ displacement of the average ΔG° could reflect the fact that some of the IDPs examined lack certain post-translational modifications that would be stabilizing to the interaction. However, the same argument may hold also for globular proteins and a phosphorylation may even destabilize a complex. The lack of other factors (chaperones, carrier proteins, methyl-groups, carbohydrates), which may alter the energy of binding *in vivo* cannot be excluded as origin for the displacement either, but again we see no reason why this should not be an even more pronounced effect for the ORD-ORD complexes.

Based on the data collected, we have reached the—perhaps—counterintuitive conclusion that interfaces formed between globular proteins and IDPs are not overall significantly different from the interfaces between two globular proteins, although the contribution of residues within the binding interface is slightly skewed. We find instead that there is a small but significant difference in the average binding free energy in favor of the ORD-ORD complexes. We suggest that this difference is primarily caused by the loss of conformational free energy upon IDP binding, which affects the off-rate of the complex, although other reasons may exists such as an increased number of binding partners for the IDP.

Finally, we would like to add that the present analysis almost exclusively involves binary complexes. It has been suggested that IDPs are particularly well suited as scaffolds for large complexes or as hubs for signaling assemblies. Therefore, we may have missed thermodynamic fingerprints that stand out and reveal IDPs that diverge more from their globular twins than the ones analyzed in the present paper. Allostery in IDP-interactions where more binding sites are in play is an emerging subject (Ferreon et al., 2013; Shammas et al., 2014) and in the ensemble view of allostery, IDP-linked negative and positive allostery is possible (Motlagh et al., 2014). This aspect is not decomposed in the present set of data and allostery may be one underlying cause of the observed differences. Also, highly fuzzy complexes acting e.g. as electrostatic clouds (Mittag et al., 2010; Fuxreiter and Tompa, 2012) are most likely not captured by the methods available for measuring the thermodynamics of protein interactions and are most definitely not targets for structure determination and hence do not contribute to the current analyses. Although the concept of fuzziness has emerged from studies on IDPs we cannot exclude that they also exist for complexes of two ordered proteins.

The reason, if any, for the evolution of protein intrinsic disorder remains to be disclosed. The present paper hints strongly that the answer does not lie directly in differences in thermodynamic parameters or the energetic principles of ligand binding.

## Author contributions

All authors contributed equally to the work and wrote the manuscript in collaboration.

### Conflict of interest statement

The authors declare that the research was conducted in the absence of any commercial or financial relationships that could be construed as a potential conflict of interest.
