# Supplementary material for: Globular and disordered—the non-identical twins in protein-protein interactions
Source: Front Mol Biosci. 2015 Jul 9;2:40. doi: 10.3389/fmolb.2015.00040 (PMC4496568; doi:10.3389/fmolb.2015.00040)
Supplement: Supplementary file 1 [file Table1.PDF]

frontiers

Supplementary Material

Globular and disordered – the non-identical twins in protein-protein interactions

Kaare Teilum*, Johan G. Olsen, Birthe B. Kragelund*

Structural Biology and NMR Laboratory, Department of Biology, University of Copenhagen, Ole Maaloes Vej 5, 2200 Copenhagen N, Denmark

*** Correspondence:**

Dr. Birthe B. Kragelund, Department of Biology, Ole Maaløes Vej 5, 2200 Copenhagen N, Denmark
bbk@bio.ku.dk

Dr. Kaare Teilum, Department of Biology, Ole Maaløes Vej 5, 2200 Copenhagen N, Denmark
kaare.teilum@bio.ku.dk

Supplementary Table 1. Thermodynamic parameters for protein-protein interactions at 25 ºC.

Protein 1	Protein 2	ΔH (kcal/mol)	TΔS (kcal/mol)	ΔG (kcal/mol)	Interface area (Å^2)	PDB code	Reference
Both interaction partners ordered							
4D5 Fab	p185-HER2	-10.40	2.66	-13.06			(Kelley and O'Connell, 1993)
Ab 13AD	peptide LZ	-12.60	-2.12	-10.48			(Leder et al., 1995)
Ab 29AB	peptide LZ	-13.80	-2.83	-10.97			(Leder et al., 1995)
Ab 2B5	cytochrome c	-21.00	-8.40	-12.60			(Raman et al., 1995)
Ab 5F8	cytochrome c	-21.70	-7.84	-13.86			(Raman et al., 1995)
Ab E3	cytochrome c	-7.30	2.62	-9.92			(Murphy et al., 1995)
Ab E8	cytochrome c	-9.50	0.09	-9.59	614	1WEJ	(Murphy et al., 1995)
Act-EF34	titin	-15.24	-5.70	-9.54			(Beck et al., 2011)
Act-EF34	titin	-15.24	-5.70	-9.54			(Beck et al., 2011)
barstar	barnase	-19.30	-0.30	-19.00	742	2ZA4	(Frisch et al., 1997)
BPTI	chymotrypsin	2.50	13.84	-11.34	770	1MTN	(Castro and Anderson, 1996)
calmodulin	seminal plasmin	0.00	8.11	-8.11			(Milos et al., 1988)
calmodulin	myosin light chain kinase	0.00	7.18	-7.18	1443	2O5G	(Milos et al., 1988)
calmodulin Ca2+	myosin light chain kinase	-20.30	-8.70	-11.60	1481	2K0F	(Milos et al., 1988)
calmodulin Ca2+	seminal plasmin	-12.00	0.00	-12.00			(Milos et al., 1988)
ch4D5 Fab	p185HER2	-17.20	-3.58	-13.62			(Kelley et al., 1992)
Che B	CheA1	-10.10	-2.59	-7.51			(Li et al., 1995)
CheY	CheA	-14.30	-6.34	-7.96	562	1A0O	(Li et al., 1995)
CheY	CheA1	-12.30	-4.29	-8.01	598	1FFG	(Li et al., 1995)
CK2alfa	CK2beta	-15.24	-4.02	-11.22	670	4NH1	(Raaf et al., 2011)
colicin N	OmpF	-12.30	-4.56	-7.74	610	3O0E	(Evans et al., 1996)

colicin N	PhoE	-6.00	1.43	-7.43			(Evans et al., 1996)
colicin N	OmpC	-3.70	3.34	-7.04			(Evans et al., 1996)
cytochrome b5	cytochrome c	1.00	10.10	-9.10			(McLean and Sligar, 1995)
cytochrome C (horse)	Cyt c peroxidase	2.25	9.18	-6.94	570	1U74	(Erman et al., 1997)
cytochrome C (yeast)	Cyt c peroxidase	-2.60	5.50	-8.10	570	1U74	(Pielak and Wang, 2001)
cytochrome C (yeast)	Cyt c peroxidase	-1.60	5.61	-7.21	570	1U74	(Volkov et al., 2009)
D1.3-mAb	E5.2-mAb	-66.70	-56.14	-10.56	934	1DVF	(Tello et al., 1994)
D1.3-mAb	hen egg white lysozyme	-21.70	-10.25	-11.45	672	1KIP	(Bhat et al., 1994)
D1.3-mAb	E225-mAb	1.80	9.06	-7.26	853	1CIC	(Tello et al., 1994)
D11.15-F_{ab}	hen egg white lysozyme	-19.00	-6.85	-12.15	839	1JHL[a]	(Schwarz et al., 1995)
D44.1-F_{ab}	hen egg white lysozyme	-10.30	-0.69	-9.61	745	1MLC	(Schwarz et al., 1995)
E2-Dnase	Im2(-Zn2+)	-38.64	-19.00	-19.64	846	3U43	(Keeble et al., 2006)
E2-Dnase	Im2(+Zn2+)	-33.09	-13.45	-19.64	846	3U43	(Keeble et al., 2006)
E7-Dnase	Im7(-Zn2+)	-56.73	-36.90	-19.83	685	7CEI	(Keeble et al., 2006)
E7-Dnase	Im7(+Zn2+)	-30.34	-10.50	-19.84	685	7CEI	(Keeble et al., 2006)
E8-Dnase	Im8(-Zn2+)	-25.84	-6.04	-19.80			(Keeble et al., 2006)
E8-Dnase	Im8(+Zn2+)	-24.23	-4.43	-19.80			(Keeble et al., 2006)
E9-Dnase	Im9 (-Zn2+)	-19.00	-0.48	-18.52	769	1BXI	(Keeble et al., 2006)
E9-Dnase	Im9 (+Zn2+)	-10.50	8.07	-18.57	769	1BXI	(Keeble et al., 2006)
Efb-C	C3d	-8.50	3.40	-11.90	829	2GOX	(Haspel et al., 2008)
elastase	ovomucoid third domain	-1.00	13.50	-14.50	661	1PPF	(Baker and Murphy, 1997)
EphB4	ephrin	3.30	13.40	-10.10	1052	2HLE	(Chrencik et al., 2006)
erythropoietin	EPO receptor site 2	-3.40	5.01	-8.41	696	1EER	(Philo et al., 1996a)
erythropoietin	EPO receptor site 1	-1.50	10.19	-11.69	975	1EER	(Philo et al., 1996a)
F9.13.7-F_{ab}	hen egg white lysozyme	-11.10	0.98	-12.08	888	1FBI[b]	(Schwarz et al., 1995)
FvD1.3	hen egg white lysozyme	-21.50	-11.04	-10.46	674	1G7M	(Sundberg et al., 2000)

FvD1.3	hen egg white lysozyme	-21.51	-10.07	-11.44	704	1A2Y	(Bhat et al., 1994)
FyHyHEL-10	hen egg white lysozyme	-21.90	-9.83	-12.07	932	2DQJ	(Shiroishi et al., 2001; Tsumoto et al., 1994)
Fyn SH3 domain	PI3	10.60	17.94	-7.34		1A0N	(Renzoni et al., 1996)
GA-BPalfa	GA-BPbeta	-10.90	0.53	-11.43	826	1AWC	(Desrosiers and Peng, 2005)
gp120	CD4	-63.00	-49.22	-13.78	975	1GC1	(Myszka et al., 2000)
Hck SH3 domain	HIV Nef protein	-12.80	-3.58	-9.22	594	3RBB	(Lee et al., 1995)
hGH-G120R	hGH-BP	-9.40	2.29	-11.69	2091	1HWG	(Pearce et al., 1996)
human tissue factor	coagulation factor VII	-32.00	-20.86	-11.14	1959	1DAN	(Kelley et al., 1995)
HyHEL-5	hen egg white lysozyme	-22.60	-8.11	-14.49	853	1YQV	(Hibbits et al., 1994)
HyHEL-5	CQL	-21.40	-6.94	-14.46			(Shick et al., 1997)
HyHEL-5	BWQL	-20.10	-10.76	-9.34			(Shick et al., 1997)
IL13	IL13Ralfa1	-11.40	-0.83	-10.57	772	3BPO	(Lupardus et al., 2010)
IL13/IL13Ralfa1	IL13Ralfa2	-20.10	-4.32	-15.78	651	3BPO	(Lupardus et al., 2010)
Interleukin 5	IL5 receptor R subunit	-11.40	0.21	-11.61	1238	3QT2	(Johanson et al., 1995)
mDia	RhoA	-2.80	7.87	-10.67			(Lammers et al., 2008)
mDia	Rac1	1.90	8.44	-6.54			(Lammers et al., 2008)
mDia	Cdc42	2.20	9.25	-7.05	1003	3EG5	(Lammers et al., 2008)
ORF158L	histone H3	-10.00	-1.21	-8.79			(Tran et al., 2011)
ORF158L	histone H3H4	-6.70	2.32	-9.02			(Tran et al., 2011)
phosphocarrier protein	enzyme I N	8.80	15.79	-6.99	975	3EZA	(Chauvin et al., 1996)
PRLR	prolactin	-25.87	-14.58	-11.29	1159	3NCB	(Hansen et al., 2011)
PSBD	E3	2.20	14.80	-12.60			(Jung et al., 2002)
R-Ras	Ral-GDS-RBD	-8.80	-0.80	-8.00			(Rudolph et al., 2001)
R-Ras	Raf-RBD	-5.60	3.10	-8.70	527	3KUD	(Rudolph et al., 2001)
Rap	Ral-GDS-RBD	-14.50	-4.70	-9.80			(Rudolph et al., 2001)
Rap	Byr2-RBD	-9.00	0.80	-9.80			(Rudolph et al., 2001)

Rap	Ras-RBD	-3.70	4.80	-8.50			(Rudolph et al., 2001)
Rap	AF6-RBD	-2.40	6.70	-9.10			(Rudolph et al., 2001)
Ras	Ral-GDS-RBD	-14.50	-6.20	-8.30			(Rudolph et al., 2001)
Ras	Byr2-RBD	-9.00	0.20	-9.20			(Rudolph et al., 2001)
Ras	AF6-RBD	-8.50	-1.00	-7.50			(Rudolph et al., 2001)
Ras	Raf-RBD	-5.20	4.50	-9.70	614	4G0N	(Rudolph et al., 2001)
Rnd1	Plexin B1	-6.70	-0.40	-7.10	671	2REX	(Wang et al., 2011)
RPP29	RPP21	5.95	15.55	-9.60	1203	2KI7	(Xu et al., 2012)
stem cell factor	Kit extracellular domain	-13.00	-3.99	-9.01	1697	2E9W	(Philo et al., 1996b)
subtilisin inhibitor	subtilisin	-4.70	9.27	-13.97			(Takahashi and Fukada, 1985)
subtilisin inhibitor	chymotrypsin	4.50	11.38	-6.88			(Fukada et al., 1985)
TC-21	Ral-GDS-RBD	-9.80	-1.10	-8.70			(Rudolph et al., 2001)
TC-21	Raf-RBD	-5.60	2.90	-8.50			(Rudolph et al., 2001)
TEM-1 b-lactamase	beta-lactamase inhib	-7.40	6.29	-13.69	1295	2B5R	(Wang et al., 2007)
trypsin	lima bean inhibitor	2.10	14.81	-12.71			(Baugh and Trowbridge, 1972)
trypsin	ovomucoid	5.60	15.82	-10.22	840	1Z7K	(Baugh and Trowbridge, 1972)
trypsin	soybean inhibitor	8.60	20.80	-12.20	870	1AVW	(Baugh and Trowbridge, 1972)
trypsin	cleaved soybean inhibitor	12.60	23.42	-10.82			(Baugh and Trowbridge, 1972)
One ordered and one disordered interaction partner							
A	B heterodimeric coiled coil	-24.70	-14.39	-10.31			(Jelesarov and Bosshard, 1996)
Ab 131	angiotensin II	-8.90	2.06	-10.96	491	3CK0	(Murphy et al., 1993)
Ab 13AD	peptide LZ(7P14P)	-17.40	-7.75	-9.65			(Leder et al., 1995)
Ab 29AB	peptide LZ(7P14P)	-17.10	-7.69	-9.41			(Leder et al., 1995)
Ab 42PF	peptide LZ(7P14P)	-13.40	-2.98	-10.42			(Leder et al., 1995)
Act-EF34	palladin	-4.50	2.09	-6.59			(Beck et al., 2011)

Bak BH3	Mcl-1	-22.10	-10.37	-11.73			(Day et al., 2008)
beta-catenin	Tcf4(1-56)	-24.50	-12.80	-11.70	1659	2GL7	(Fasolini et al., 2003)
beta-catenin	p-APC-R3	-22.50	-10.78	-11.72	2520	1TH1	(Liu et al., 2006)
beta-catenin	APC-R3	-16.80	-7.93	-8.87		1T08	(Liu et al., 2006)
beta-catenin	APC-R3-long	-15.50	-9.26	-6.24			(Tickenbrock et al., 2003)
beta-catenin	APC-R4	-2.60	3.46	-6.06			(Tickenbrock et al., 2003)
Bim BH3	Mcl-1	-19.20	-7.18	-12.02	997	2PQK	(Day et al., 2008)
Bmf BH3	Mcl-1	-11.50	-2.65	-8.85			(Day et al., 2008)
calmodulin	smMLCKp	-29.79	-18.03	-11.76			(Frederick et al., 2006)
calmodulin	CaMKIp	-28.43	-16.09	-12.34			(Frederick et al., 2006)
calmodulin	NOS-1	-7.26	2.58	-9.84	1284	2O60	(Censarek et al., 2002)
calmodulin	cNOS	-2.80	8.30	-11.10			(Brokx et al., 2001)
calmodulin	PDE	3.80	14.75	-10.95			(Brokx et al., 2001)
calmodulin	MLC	3.99	13.13	-9.14			(Brokx et al., 2001)
calmodulin	melittin	4.80	12.90	-8.10			(Milos et al., 1987)
calmodulinCa2+	melittin	7.20	18.86	-11.66			(Milos et al., 1987)
Cav1-alfa AID	Cavbeta ABP	-15.80	-4.98	-10.82			(Van Petegem et al., 2008)
CcdA	CcdB_2	-35.50	-19.90	-15.60	1373	3TCJ	(Drobnak et al., 2013)
CcdA	CcdB_2-CcdA	-15.00	-7.61	-7.39	1373	do	(Drobnak et al., 2013)
Cdk4/cyclin A	p27	-49.10	-37.80	-11.30	1104	1JSU	(Ou et al., 2011)
Cdk4/cyclinD	p27	-34.40	-22.70	-11.70			(Ou et al., 2011)
CheR	receptor pentapeptide	-13.60	-5.63	-7.97	503	1BC5	(Wu et al., 1996)
CP12-2	GAPDH	-15.00	-4.92	-10.08	2050	3B1K	(Marri et al., 2008)
E3-ligase EloB/EloC	Vif	-20.20	-7.30	-12.90	629	4N9F	(Wolfe et al., 2010)
endothiapepsin	pepstatin A	-2.50	6.79	-9.29	634	4ER2	(Gómez and Freire, 1995)
ERK2	STEP	-29.50	-19.70	-9.80			(Francis et al., 2014)

ERK2	DUSP16 MKBD	-4.80	3.20	-8.00			(Francis et al., 2014)
ERRgamma LBD	PGC-1alfa	-5.40	2.75	-8.15			(Devarakonda et al., 2011)
FBP21 Tandem WW	SMB peptide 4	-15.53	-9.37	-6.16			(Klippel et al., 2011)
FBP21 Tandem WW	SMB peptide 2	-8.84	-2.52	-6.32			(Klippel et al., 2011)
FBP21 Tandem WW	SF3B4 peptide 2	-7.17	-1.52	-5.65			(Klippel et al., 2011)
FBP21 Tandem WW	SMB peptide 1	-5.01	-0.51	-4.50			(Klippel et al., 2011)
FBP21 Tandem WW	SF3B4 peptide 1	-1.91	2.28	-4.19			(Klippel et al., 2011)
FN NTD	Sfbl-5	-45.50	-32.18	-13.32	980	3ZRZ	(Norris et al., 2011)
Fyn SH2 domain	pY531 phosphopeptide	-8.70	-0.39	-8.31			(Ladbury et al., 1996)
Fyn SH2 domain	pYHmT phosphopeptide	-4.30	1.97	-6.27			(Ladbury et al., 1996)
Fyn SH3 domain	P2L peptide	-12.30	-5.54	-6.76	376		(Renzoni et al., 1996)
Grb SH2 domain	Shc peptide	-7.94	1.18	-9.12			(McNemar et al., 1997)
Grb-cSH3	SOS-1 peptide	-4.45	0.88	-5.33			(McDonald et al., 2009)
Grb-nSH3	SOS-1 peptide	-9.41	-3.39	-6.02	463	3GBQ	(McDonald et al., 2009)
Grb2-SH3C	Gab2b peptide	-11.8	-4.46	-7.34			(Krieger et al., 2014)
GroEL	unfolded subtilisin BPN′ mutant	19.90	28.52	-8.62			(Lin et al., 1995)
hGrb2 (SH3)	hSos peptide	-6.20	0.21	-6.41	411	1AZE	(Lemmon et al., 1994)
HIVgp120 V3	mAB447-52D	-8.54	1.88	-10.42			(Killikelly et al., 2013)
hSBD6	hF3	6.20	13.63	-7.43	629	3RNM	(Brautigam et al., 2011)
hsRAd51	BRC-pep	-12.30	-5.36	-6.94	1041	1N0W	(Nomme et al., 2010)
IC	Tctex1	-6.90	-0.10	-6.80	2189	3FM7	(Hall et al., 2009)
IC	LC8	1.10	8.10	-7.00	896	3FM7/3GLW	(Hall et al., 2009)
IKBalfa	Rel-A	-3.70	4.48	-8.18			(Cervantes et al., 2011)
IRS1 PTB	hEGFR	-2.82	5.02	-7.84			(Farooq et al., 1999)
IRS1 PTB	hErbB3	-2.54	6.27	-8.81			(Farooq et al., 1999)

IRS1 PTB	IL-4R	0.87	7.58	-6.71	682	1IRS	(Farooq et al., 1999)
IRS1 PTB	IR	2.54	9.85	-7.31			(Farooq et al., 1999)
IRS1 PTB	TrkA pep	3.63	12.75	-9.12			(Farooq et al., 1999)
Keap1	Neh2	-28.40	-17.10	-11.30	498	3ZGC	(Tong et al., 2006)
Keap1	Neh2 site 2	-11.20	-3.00	-8.20			(Tong et al., 2006)
Kelch domain	NRF2	-16.96	-6.56	-10.40	748	3WN7	(Cino et al., 2013)
KIX	MLL	-14.90	-6.88	-8.02	815	2LXS	(Arai et al., 2010)
Lck SH2 domain	Lck phosphopeptide	-8.40	-1.04	-7.36	396	1CWD	(Ladbury et al., 1995)
MDM2	p53(17-26)	-12.30	-2.84	-9.46			(Schon et al., 2002)
MDM2	p73(11-22)	-9.50	-0.87	-8.63			(Schon et al., 2002)
MDM2	p53(15-29)	-6.60	1.75	-8.35			(Schon et al., 2002)
mNoxaB BH3	Mcl-1	-23.60	-13.89	-9.71	808	2NLA	(Day et al., 2008)
NCBD	ACTR	-31.70	-20.88	-10.82	1655	1KBH	(Demarest et al., 2004)
NCBD	p53(38-61)	-4.60	2.17	-6.77			(Lee et al., 2010)
NCBD	p53(13-61)	-4.42	3.24	-7.66	1115	2L14	(Lee et al., 2010)
NCBD	p53(25-61)	-4.12	3.19	-7.31			(Lee et al., 2010)
NoxaB BH3	Mcl-1	-32.60	-23.21	-9.39	1095	2JM6	(Day et al., 2008)
NoxaA BH3	Mcl-1	-20.70	-10.43	-10.27	1093	2ROD	(Day et al., 2008)
p85 SH2 domain	PDGFR phosphopeptide	-9.40	-0.72	-8.68	646	2IUI	(Ladbury et al., 1995)
PCNA	p21(141-160)	-9.58	0.03	-9.61	710		(Zheleva et al., 2000)
profilin	Pro11	-5.10	0.33	-5.43	694	1AWI[c]	(Petrella et al., 1996)
PSD-95 PDZs	Neurolignin-1 peptide	-8.90	-2.10	-6.80			(Saro et al., 2007)
PSD-95 PDZs	Cript peptide	-6.40	1.40	-7.80	379		(Saro et al., 2007)
PSD-95 PDZs	Citron-based peptide	-6.10	-0.30	-5.80			(Saro et al., 2007)
Puma BH3	Mcl-1	-24.50	-12.61	-11.89	983	2ROC	(Day et al., 2008)
Rab11a	FIP2	-23.50	-15.50	-8.00	599	4C4P	(Junutula et al., 2004)

Rab11a	RCP	-16.70	-8.60	-8.10			(Junutula et al., 2004)
Rab11a	Rip11	-12.80	-4.60	-8.20			(Junutula et al., 2004)
RST	ANAC013	-9.79	-1.29	-8.50			(O'Shea et al., 2015)
RST	ANAC046	-4.37	4.11	-8.48			(O'Shea et al., 2015)
S-protein	S-peptide (4-14)	-32.5	-24.7	-7.80	608	1D5E	(Ratnaparkhi and Varadarajan, 2000)
S-protein	S peptide (1-15)	-41.90	-32.48	-9.42	650	1RBC	(Varadarajan et al., 1992)
SCH PTB	EFGR	-5.46	4.84	-10.30			(Mandiyan et al., 1996)
SCH PTB	TrkA pep	2.36	12.44	-10.08			(Mandiyan et al., 1996)
Sem-SH3	SOS-1 peptide	-7.92	-1.85	-6.07	373	1SEM	(Hamburger et al., 2004)
SHC N-term. Domain	EGFR1148 peptide	-5.46	4.83	-10.29			(Mandiyan et al., 1996)
SHC N-term. Domain	Trk490 peptide	2.40	12.43	-10.03			(Mandiyan et al., 1996)
Spc SH3 domain	p41 peptide	-10.86	-5.86	-5.00			(Casares et al., 2007)
Src SH2 domain	pYHmT phosphopeptide	-8.40	0.12	-8.52			(Ladbury et al., 1995)
Src SH2 domain	pYEE1	-7.70	1.50	-9.20			(Bradshaw et al., 1999)
Src SH2 domain	hmT peptide	-6.50	2.60	-9.10			(Bradshaw et al., 1998)
Src SH2 domain	C-tail	-4.60	1.60	-6.20			(Bradshaw et al., 1998)
Src SH2 domain	PDGFR phosphopeptide	-3.40	3.70	-7.10			(Bradshaw et al., 1998)
streptavidin	FSHPQNT peptide	-19.30	-14.01	-5.29	439	1VWA	(Weber et al., 1992)
streptavidin	pStrep-tag	-12.60	-6.50	-6.10	504	1RSU	(Schmidt et al., 1996)
YAP2 WW1	WBP1_PY2	-11.59	-6.29	-5.30			(McDonald et al., 2011)
YAP2 WW1	WBP1_PY1	-2.39	2.38	-4.77			(McDonald et al., 2011)
YAP2 WW2	WBP1_PY2	-14.13	-9.06	-5.07			(McDonald et al., 2011)
YAP2 WW2	WBP1_PY1	-4.31	0.77	-5.08			(McDonald et al., 2011)

[a]Structure solved with pheasant lysozyme
[b]Structure solved with guinea fowl lysozyme
[c]Structure solved with Pro_{10} peptide

References

Arai, M., Dyson, H. J., and Wright, P. E. (2010). Leu628 of the KIX domain of CBP is a key residue for the interaction with the MLL transactivation domain. *FEBS Lett.* 584, 4500–4504. doi:10.1016/j.febslet.2010.10.024.

Baker, B. M., and Murphy, K. P. (1997). Dissecting the energetics of a protein-protein interaction: the binding of ovomucoid third domain to elastase. *J. Mol. Biol.* 268, 557–569. doi:10.1006/jmbi.1997.0977.

Baugh, R. J., and Trowbridge, C. G. (1972). Calorimetry of some trypsin-trypsin inhibitor reactions. *J. Biol. Chem.* 247, 7498–7501.

Beck, M. R., Otey, C. A., and Campbell, S. L. (2011). Structural characterization of the interactions between palladin and α-actinin. *J. Mol. Biol.* 413, 712–725. doi:10.1016/j.jmb.2011.08.059.

Bhat, T. N., Bentley, G. A., Boulot, G., Greene, M. I., Tello, D., Dall'Acqua, W., Souchon, H., Schwarz, F. P., Mariuzza, R. A., and Poljak, R. J. (1994). Bound water molecules and conformational stabilization help mediate an antigen-antibody association. *Proc. Natl. Acad. Sci. U.S.A.* 91, 1089–1093.

Bradshaw, J. M., Grucza, R. A., Ladbury, J. E., and Waksman, G. (1998). Probing the "two-pronged plug two-holed socket" model for the mechanism of binding of the Src SH2 domain to phosphotyrosyl peptides: A thermodynamic study. *Biochemistry* 37, 9083–9090. doi:10.1021/bi973147k.

Bradshaw, J. M., Mitaxov, V., and Waksman, G. (1999). Investigation of phosphotyrosine recognition by the SH2 domain of the Src kinase. *J. Mol. Biol.* 293, 971–985. doi:10.1006/jmbi.1999.3190.

Brautigam, C. A., Wynn, R. M., Chuang, J. L., Naik, M. T., Young, B. B., Huang, T.-H., and Chuang, D. T. (2011). Structural and Thermodynamic Basis for Weak Interactions between Dihydrolipoamide Dehydrogenase and Subunit-binding Domain of the Branched-chain alpha-Ketoacid Dehydrogenase Complex. *J. Biol. Chem.* 286, 23476–23488. doi:10.1074/jbc.M110.202960.

Brokx, R. D., Lopez, M. M., Vogel, H. J., and Makhatadze, G. I. (2001). Energetics of Target Peptide Binding by Calmodulin Reveals Different Modes of Binding. *J. Biol. Chem.* 276, 14083–14091.

Casares, S., AB, E., Eshuis, H., Lopez-Mayorga, O., van Nuland, N. A., and Conejero-Lara, F. (2007). The high-resolution NMR structure of the R21A Spc-SH3:P41 complex: Understanding the determinants of binding affinity by comparison with Abl-SH3. *BMC Struct. Biol.* 7, 22–19. doi:10.1186/1472-6807-7-22.

Castro, M. J., and Anderson, S. (1996). Alanine point-mutations in the reactive region of bovine pancreatic trypsin inhibitor: effects on the kinetics and thermodynamics of binding to beta-trypsin and alpha-chymotrypsin. *Biochemistry* 35, 11435–

11446. doi:10.1021/bi960515w.

Censarek, P., Beyermann, M., and Koch, K.-W. (2002). Target recognition of apocalmodulin by nitric oxide synthase I peptides. *Biochemistry* 41, 8598–8604. doi:10.1021/bi025681k.

Cervantes, C. F., Bergqvist, S., Kjaergaard, M., Kroon, G., Sue, S.-C., Dyson, H. J., and Komives, E. A. (2011). The RelA nuclear localization signal folds upon binding to IκBα. *J. Mol. Biol.* 405, 754–764. doi:10.1016/j.jmb.2010.10.055.

Chauvin, F., Fomenkov, A., Johnson, C. R., and Roseman, S. (1996). The N-terminal domain of Escherichia coli enzyme I of the phosphoenolpyruvate/glycose phosphotransferase system: molecular cloning and characterization. *Proc. Natl. Acad. Sci. U.S.A.* 93, 7028–7031.

Chrencik, J. E., Brooun, A., Kraus, M. L., Recht, M. I., Kolatkar, A. R., Han, G. W., Seifert, J. M., Widmer, H., Auer, M., and Kuhn, P. (2006). Structural and biophysical characterization of the EphB4*ephrinB2 protein-protein interaction and receptor specificity. *J. Biol. Chem.* 281, 28185–28192. doi:10.1074/jbc.M605766200.

Cino, E. A., Killoran, R. C., Karttunen, M., and Choy, W.-Y. (2013). Binding of disordered proteins to a protein hub. *Sci Rep* 3, 2305. doi:10.1038/srep02305.

Day, C. L., Smits, C., Fan, F. C., Lee, E. F., Fairlie, W. D., and Hinds, M. G. (2008). Structure of the BH3 domains from the p53-inducible BH3-only proteins Noxa and Puma in complex with Mcl-1. *J. Mol. Biol.* 380, 958–971. doi:10.1016/j.jmb.2008.05.071.

Demarest, S. J., Deechongkit, S., Dyson, H. J., Evans, R. M., and Wright, P. E. (2004). Packing, specificity, and mutability at the binding interface between the p160 coactivator and CREB-binding protein. *Protein Sci.* 13, 203–210. doi:10.1110/ps.03366504.

Desrosiers, D. C., and Peng, Z.-Y. (2005). A binding free energy hot spot in the ankyrin repeat protein GABPbeta mediated protein-protein interaction. *J. Mol. Biol.* 354, 375–384. doi:10.1016/j.jmb.2005.09.045.

Devarakonda, S., Gupta, K., Chalmers, M. J., Hunt, J. F., Griffin, P. R., Van Duyne, G. D., and Spiegelman, B. M. (2011). Disorder-to-order transition underlies the structural basis for the assembly of a transcriptionally active PGC-1α/ERRγ complex. *Proc. Natl. Acad. Sci. U.S.A.* 108, 18678–18683. doi:10.1073/pnas.1113813108.

Drobnak, I., De Jonge, N., Haesaerts, S., Vesnaver, G., Loris, R., and Lah, J. (2013). Energetic basis of uncoupling folding from binding for an intrinsically disordered protein. *J. Am. Chem. Soc.* 135, 1288–1294. doi:10.1021/ja305081b.

Erman, J. E., Kresheck, G. C., Vitello, L. B., and Miller, M. A. (1997). Cytochrome c/cytochrome c peroxidase complex: effect of binding-site mutations on the thermodynamics of complex formation. *Biochemistry* 36, 4054–4060.

doi:10.1021/bi962632x.

Evans, L. J., Cooper, A., and Lakey, J. H. (1996). Direct measurement of the association of a protein with a family of membrane receptors. *J. Mol. Biol.* 255, 559–563. doi:10.1006/jmbi.1996.0047.

Farooq, A., Plotnikova, O., Zeng, L., and Zhou, M. M. (1999). Phosphotyrosine binding domains of Shc and insulin receptor substrate 1 recognize the NPXpY motif in a thermodynamically distinct manner. *J. Biol. Chem.* 274, 6114–6121.

Fasolini, M., Wu, X., Flocco, M., Trosset, J.-Y., Oppermann, U., and Knapp, S. (2003). Hot spots in Tcf4 for the interaction with beta-catenin. *J. Biol. Chem.* 278, 21092–21098. doi:10.1074/jbc.M301781200.

Francis, D. M., Koveal, D., Tortajada, A., Page, R., and Peti, W. (2014). Interaction of kinase-interaction-motif protein tyrosine phosphatases with the mitogen-activated protein kinase ERK2. *PLoS ONE* 9, e91934. doi:10.1371/journal.pone.0091934.

Frederick, K. K., Kranz, J. K., and Wand, A. J. (2006). Characterization of the backbone and side chain dynamics of the CaM-CaMKIp complex reveals microscopic contributions to protein conformational entropy. *Biochemistry* 45, 9841–9848. doi:10.1021/bi060865a.

Frisch, C., Schreiber, G., Johnson, C. M., and Fersht, A. R. (1997). Thermodynamics of the interaction of barnase and barstar: changes in free energy versus changes in enthalpy on mutation. *J. Mol. Biol.* 267, 696–706. doi:10.1006/jmbi.1997.0892.

Fukada, H., Takahashi, K., and Sturtevant, J. M. (1985). Thermodynamics of the binding of Streptomyces subtilisin inhibitor to alpha-chymotrypsin. *Biochemistry* 24, 5109–5115.

Gómez, J., and Freire, E. (1995). Thermodynamic mapping of the inhibitor site of the aspartic protease endothiapepsin. *J. Mol. Biol.* 252, 337–350. doi:10.1006/jmbi.1995.0501.

Hall, J., Karplus, P. A., and Barbar, E. (2009). Multivalency in the assembly of intrinsically disordered Dynein intermediate chain. *J. Biol. Chem.* 284, 33115–33121. doi:10.1074/jbc.M109.048587.

Hamburger, J. B., Ferreon, J. C., Whitten, S. T., and Hilser, V. J. (2004). Thermodynamic mechanism and consequences of the polyproline II (PII) structural bias in the denatured states of proteins. *Biochemistry* 43, 9790–9799. doi:10.1021/bi049352z.

Hansen, M. J. K., Olsen, J. G., Bernichtein, S., O'Shea, C., Sigurskjold, B. W., Goffin, V., and Kragelund, B. B. (2011). Development of prolactin receptor antagonists with reduced pH-dependence of receptor binding. *J. Mol. Recognit.* 24, 533–547. doi:10.1002/jmr.1064.

Haspel, N., Ricklin, D., Geisbrecht, B. V., Kavraki, L. E., and Lambris, J. D. (2008). Electrostatic contributions drive the interaction between Staphylococcus aureus

protein Efb-C and its complement target C3d. *Protein Science* 17, 1894–1906. doi:10.1110/ps.036624.108.

Hibbits, K. A., Gill, D. S., and Willson, R. C. (1994). Isothermal titration calorimetric study of the association of hen egg lysozyme and the anti-lysozyme antibody HyHEL-5. *Biochemistry* 33, 3584–3590.

Jelesarov, I., and Bosshard, H. R. (1996). Thermodynamic characterization of the coupled folding and association of heterodimeric coiled coils (leucine zippers). *J. Mol. Biol.* 263, 344–358. doi:10.1006/jmbi.1996.0579.

Johanson, K., Appelbaum, E., Doyle, M., Hensley, P., Zhao, B., Abdel-Meguid, S. S., Young, P., Cook, R., Carr, S., and Matico, R. (1995). Binding interactions of human interleukin 5 with its receptor alpha subunit. Large scale production, structural, and functional studies of Drosophila-expressed recombinant proteins. *J. Biol. Chem.* 270, 9459–9471.

Jung, H. I., Cooper, A., and Perham, R. N. (2002). Identification of key amino acid residues in the assembly of enzymes into the pyruvate dehydrogenase complex of Bacillus stearothermophilus: A kinetic and thermodynamic analysis. *Biochemistry* 41, 10446–10453. doi:10.1021/bi020147y.

Junutula, J. R., Schonteich, E., Wilson, G. M., Peden, A. A., Scheller, R. H., and Prekeris, R. (2004). Molecular characterization of Rab11 interactions with members of the family of Rab11-interacting proteins. *J. Biol. Chem.* 279, 33430–33437. doi:10.1074/jbc.M404633200.

Keeble, A. H., Kirkpatrick, N., Shimizu, S., and Kleanthous, C. (2006). Calorimetric dissection of colicin DNase--immunity protein complex specificity. *Biochemistry* 45, 3243–3254. doi:10.1021/bi052373o.

Kelley, R. F., and O'Connell, M. P. (1993). Thermodynamic Analysis of an Antibody Functional Epitope. *Biochemistry* 32, 6828–6835.

Kelley, R. F., Costas, K. E., O'Connell, M. P., and Lazarus, R. A. (1995). Analysis of the Factor Viia Binding-Site on Human Tissue Factor - Effects of Tissue Factor Mutations on the Kinetics and Thermodynamics of Binding. *Biochemistry* 34, 10383–10392. doi:10.1021/bi00033a009.

Kelley, R. F., O'Connell, M. P., Carter, P., Presta, L., Eigenbrot, C., Covarrubias, M., Snedecor, B., Bourell, J. H., and Vetterlein, D. (1992). Antigen binding thermodynamics and antiproliferative effects of chimeric and humanized anti-p185HER2 antibody Fab fragments. *Biochemistry* 31, 5434–5441.

Killikelly, A., Zhang, H.-T., Spurrier, B., Williams, C., Gorny, M. K., Zolla-Pazner, S., and Kong, X.-P. (2013). Thermodynamic signatures of the antigen binding site of mAb 447-52D targeting the third variable region of HIV-1 gp120. *Biochemistry* 52, 6249–6257. doi:10.1021/bi400645e.

Klippel, S., Wieczorek, M., Schümann, M., Krause, E., Marg, B., Seidel, T., Meyer, T., Knapp, E.-W., and Freund, C. (2011). Multivalent binding of formin-binding

protein 21 (FBP21)-tandem-WW domains fosters protein recognition in the pre-spliceosome. *J. Biol. Chem.* 286, 38478–38487. doi:10.1074/jbc.M111.265710.

Krieger, J. M., Fusco, G., Lewitzky, M., Simister, P. C., Marchant, J., Camilloni, C., Feller, S. M., and De Simone, A. (2014). Conformational recognition of an intrinsically disordered protein. *Biophys. J.* 106, 1771–1779. doi:10.1016/j.bpj.2014.03.004.

Ladbury, J. E., Hensmann, M., Panayotou, G., and Campbell, I. D. (1996). Alternative modes of tyrosyl phosphopeptide binding to a Src family SH2 domain: implications for regulation of tyrosine kinase activity. *Biochemistry* 35, 11062–11069. doi:10.1021/bi960543e.

Ladbury, J. E., Lemmon, M. A., Zhou, M., Green, J., Botfield, M. C., and Schlessinger, J. (1995). Measurement of the binding of tyrosyl phosphopeptides to SH2 domains: a reappraisal. *Proc. Natl. Acad. Sci. U.S.A.* 92, 3199–3203.

Lammers, M., Meyer, S., Kuehlmann, D., and Wittinghofer, A. (2008). Specificity of Interactions between mDia Isoforms and Rho Proteins. *J. Biol. Chem.* 283, 35236–35246. doi:10.1074/jbc.M805634200.

Leder, L., Berger, C., Bornhauser, S., Wendt, H., Ackermann, F., Jelesarov, I., and Bosshard, H. R. (1995). Spectroscopic, calorimetric, and kinetic demonstration of conformational adaptation in peptide-antibody recognition. *Biochemistry* 34, 16509–16518.

Lee, C. H., Leung, B., Lemmon, M. A., Zheng, J., Cowburn, D., Kuriyan, J., and Saksela, K. (1995). A single amino acid in the SH3 domain of Hck determines its high affinity and specificity in binding to HIV-1 Nef protein. *EMBO J.* 14, 5006–5015.

Lee, C. W., Martinez-Yamout, M. A., Dyson, H. J., and Wright, P. E. (2010). Structure of the p53 transactivation domain in complex with the nuclear receptor coactivator binding domain of CREB binding protein. *Biochemistry* 49, 9964–9971. doi:10.1021/bi1012996.

Lemmon, M. A., Ladbury, J. E., Mandiyan, V., Zhou, M., and Schlessinger, J. (1994). Independent binding of peptide ligands to the SH2 and SH3 domains of Grb2. *J. Biol. Chem.* 269, 31653–31658.

Li, J. Y., Swanson, R. V., Simon, M. I., and Weis, R. M. (1995). The Response Regulators Cheb and Chey Exhibit Competitive-Binding to the Kinase Chea. *Biochemistry* 34, 14626–14636. doi:10.1021/bi00045a003.

Lin, Z., Schwartz, F. P., and Eisenstein, E. (1995). The hydrophobic nature of GroEL-substrate binding. *J. Biol. Chem.* 270, 1011–1014.

Liu, J., Xing, Y., Hinds, T. R., Zheng, J., and Xu, W. (2006). The third 20 amino acid repeat is the tightest binding site of APC for beta-catenin. *J. Mol. Biol.* 360, 133–144. doi:10.1016/j.jmb.2006.04.064.

Lupardus, P. J., Birnbaum, M. E., and Garcia, K. C. (2010). Molecular basis for

shared cytokine recognition revealed in the structure of an unusually high affinity complex between IL-13 and IL-13Ralpha2. *Structure* 18, 332–342. doi:10.1016/j.str.2010.01.003.

Mandiyan, V., O'Brien, R., Zhou, M., Margolis, B., Lemmon, M. A., Sturtevant, J. M., and Schlessinger, J. (1996). Thermodynamic studies of SHC phosphotyrosine interaction domain recognition of the NPXpY motif. *J. Biol. Chem.* 271, 4770–4775.

Marri, L., Trost, P., Trivelli, X., Gonnelli, L., Pupillo, P., and Sparla, F. (2008). Spontaneous assembly of photosynthetic supramolecular complexes as mediated by the intrinsically unstructured protein CP12. *J. Biol. Chem.* 283, 1831–1838. doi:10.1074/jbc.M705650200.

McDonald, C. B., McIntosh, S. K. N., Mikles, D. C., Bhat, V., Deegan, B. J., Seldeen, K. L., Saeed, A. M., Buffa, L., Sudol, M., Nawaz, Z., et al. (2011). Biophysical analysis of binding of WW domains of the YAP2 transcriptional regulator to PPXY motifs within WBP1 and WBP2 adaptors. *Biochemistry* 50, 9616–9627. doi:10.1021/bi201286p.

McDonald, C. B., Seldeen, K. L., Deegan, B. J., and Farooq, A. (2009). SH3 domains of Grb2 adaptor bind to PXpsiPXR motifs within the Sos1 nucleotide exchange factor in a discriminate manner. *Biochemistry* 48, 4074–4085. doi:10.1021/bi802291y.

McLean, M. A., and Sligar, S. G. (1995). Thermodynamic characterization of the interaction between cytochrome b5 and cytochrome c. *Biochem. Biophys. Res. Commun.* 215, 316–320.

McNemar, C., Snow, M. E., Windsor, W. T., Prongay, A., Mui, P., Zhang, R. M., Durkin, J., Le, H. V., and Weber, P. C. (1997). Thermodynamic and structural analysis of phosphotyrosine polypeptide binding to Grb2-SH2. *Biochemistry* 36, 10006–10014. doi:10.1021/bi9704360.

Milos, M., Schaer, J. J., Comte, M., and Cox, J. A. (1988). Microcalorimetric investigation of the interaction of calmodulin with seminalplasmin and myosin light chain kinase. *J. Biol. Chem.* 263, 9218–9222.

Milos, M., Schaer, J. J., Comte, M., and Cox, J. A. (1987). Microcalorimetric investigation of the interactions in the ternary complex calmodulin-calcium-melittin. *J. Biol. Chem.* 262, 2746–2749.

Murphy, K. P., Freire, E., and Paterson, Y. (1995). Configurational effects in antibody-antigen interactions studied by microcalorimetry. *Proteins* 21, 83–90. doi:10.1002/prot.340210202.

Murphy, K. P., Xie, D., Garcia, K. C., Amzel, L. M., and Freire, E. (1993). Structural energetics of peptide recognition: angiotensin II/antibody binding. *Proteins* 15, 113–120. doi:10.1002/prot.340150203.

Myszka, D. G., Sweet, R. W., Hensley, P., Brigham-Burke, M., Kwong, P. D.,

Hendrickson, W. A., Wyatt, R., Sodroski, J., and DOYLE, M. L. (2000). Energetics of the HIV gp120-CD4 binding reaction. *Proc. Natl. Acad. Sci. U.S.A.* 97, 9026–9031.

Nomme, J., Renodon-Cornière, A., Asanomi, Y., Sakaguchi, K., Stasiak, A. Z., Stasiak, A., Norden, B., Tran, V., and Takahashi, M. (2010). Design of potent inhibitors of human RAD51 recombinase based on BRC motifs of BRCA2 protein: modeling and experimental validation of a chimera peptide. *J. Med. Chem.* 53, 5782–5791. doi:10.1021/jm1002974.

Norris, N. C., Bingham, R. J., Harris, G., Speakman, A., Jones, R. P. O., Leech, A., Turkenburg, J. P., and Potts, J. R. (2011). Structural and functional analysis of the tandem β-zipper interaction of a Streptococcal protein with human fibronectin. *J. Biol. Chem.* 286, 38311–38320. doi:10.1074/jbc.M111.276592.

O'Shea, C., Kryger, M., Stender, E. G. P., Kragelund, B. B., Willemoës, M., and Skriver, K. (2015). Protein intrinsic disorder in Arabidopsis NAC transcription factors: transcriptional activation by ANAC013 and ANAC046 and their interactions with RCD1. *Biochem. J.* 465, 281–294. doi:10.1042/BJ20141045.

Ou, L., Ferreira, A. M., Otieno, S., Xiao, L., Bashford, D., and Kriwacki, R. W. (2011). Incomplete folding upon binding mediates Cdk4/cyclin D complex activation by tyrosine phosphorylation of inhibitor p27 protein. *J. Biol. Chem.* 286, 30142–30151. doi:10.1074/jbc.M111.244095.

Pearce, K. H., Ultsch, M. H., Kelley, R. F., deVos, A. M., and Wells, J. A. (1996). Structural and mutational analysis of affinity-inert contact residues at the growth hormone-receptor interface. *Biochemistry* 35, 10300–10307. doi:10.1021/bi960513b.

Petrella, E. C., Machesky, L. M., Kaiser, D. A., and Pollard, T. D. (1996). Structural requirements and thermodynamics of the interaction of proline peptides with profilin. *Biochemistry* 35, 16535–16543. doi:10.1021/bi961498d.

Philo, J. S., Aoki, K. H., Arakawa, T., Narhi, L. O., and Wen, J. (1996a). Dimerization of the extracellular domain of the erythropoietin (EPO) receptor by EPO: one high-affinity and one low-affinity interaction. *Biochemistry* 35, 1681–1691. doi:10.1021/bi9524272.

Philo, J. S., Wen, J., Wypych, J., Schwartz, M. G., Mendiaz, E. A., and Langley, K. E. (1996b). Human stem cell factor dimer forms a complex with two molecules of the extracellular domain of its receptor, Kit. *J. Biol. Chem.* 271, 6895–6902.

Pielak, G. J., and Wang, X. M. (2001). Interactions between yeast iso-1-cytochrome c and its peroxidase. *Biochemistry* 40, 422–428. doi:10.1021/bi002124u.

Raaf, J., Bischoff, N., Klopffleisch, K., Brunstein, E., Olsen, B. B., Vilk, G., Litchfield, D. W., Issinger, O.-G., and Niefind, K. (2011). Interaction between CK2α and CK2β, the subunits of protein kinase CK2: thermodynamic contributions of key residues on the CK2α surface. *Biochemistry* 50, 512–522. doi:10.1021/bi1013563.

Raman, C. S., Allen, M. J., and Nall, B. T. (1995). Enthalpy of antibody--cytochrome c binding. *Biochemistry* 34, 5831–5838.

Ratnaparkhi, G. S., and Varadarajan, R. (2000). Thermodynamic and structural studies of cavity formation in proteins suggest that loss of packing interactions rather than the hydrophobic effect dominates the observed energetics. *Biochemistry* 39, 12365–12374. doi:10.1021/bi000775k.

Renzoni, D. A., Pugh, D. J., Siligardi, G., Das, P., Morton, C. J., Rossi, C., Waterfield, M. D., Campbell, I. D., and Ladbury, J. E. (1996). Structural and thermodynamic characterization of the interaction of the SH3 domain from Fyn with the proline-rich binding site on the p85 subunit of PI3-kinase. *Biochemistry* 35, 15646–15653. doi:10.1021/bi9620969.

Rudolph, M. G., Linnemann, T., Grunewald, P., Wittinghofer, A., Vetter, I. R., and Herrmann, C. (2001). Thermodynamics of Ras/effector and Cdc42/effector interactions probed by isothermal titration calorimetry. *J. Biol. Chem.* 276, 23914–23921. doi:10.1074/jbc.M011600200.

Saro, D., Li, T., Rupasinghe, C., Paredes, A., Caspers, N., and Spaller, M. R. (2007). A Thermodynamic Ligand Binding Study of the Third PDZ Domain (PDZ3) from the Mammalian Neuronal Protein PSD-95 †. *Biochemistry* 46, 6340–6352. doi:10.1021/bi062088k.

Schmidt, T. G., Koepke, J., Frank, R., and Skerra, A. (1996). Molecular interaction between the Strep-tag affinity peptide and its cognate target, streptavidin. *J. Mol. Biol.* 255, 753–766. doi:10.1006/jmbi.1996.0061.

Schon, O., Friedler, A., Bycroft, M., Freund, S. M. V., and Fersht, A. R. (2002). Molecular mechanism of the interaction between MDM2 and p53. *J. Mol. Biol.* 323, 491–501.

Schwarz, F. P., Tello, D., Goldbaum, F. A., Mariuzza, R. A., and Poljak, R. J. (1995). Thermodynamics of antigen-antibody binding using specific anti-lysozyme antibodies. *Eur J Biochem* 228, 388–394.

Shick, K. A., Xavier, K. A., Rajpal, A., SmithGill, S. J., and Willson, R. C. (1997). Association of the anti-hen egg lysozyme antibody HyHEL-5 with avian species variant and mutant lysozymes. *Biochim. Biophys. Acta* 1340, 205–214.

Shiroishi, M., Yokota, A., Tsumoto, K., Kondo, H., Nishimiya, Y., Horii, K., Matsushima, M., Ogasahara, K., Yutani, K., and Kumagai, I. (2001). Structural evidence for entropic contribution of salt bridge formation to a protein antigen-antibody interaction: the case of hen lysozyme-HyHEL-10 Fv complex. *J. Biol. Chem.* 276, 23042–23050. doi:10.1074/jbc.M100480200.

Sundberg, E. J., Urrutia, M., Braden, B. C., Isern, J., Tsuchiya, D., Fields, B. A., Malchiodi, E. L., Tormo, J., Schwarz, F. P., and Mariuzza, R. A. (2000). Estimation of the hydrophobic effect in an antigen-antibody protein-protein interface. *Biochemistry* 39, 15375–15387. doi:10.1021/bi000704l.

Takahashi, K., and Fukada, H. (1985). Calorimetric studies of the binding of Streptomyces subtilisin inhibitor to subtilisin of Bacillus subtilis strain N'. *Biochemistry* 24, 297–300.

Tello, D., Eisenstein, E., Schwarz, F. P., Goldbaum, F. A., Fields, B. A., Mariuzza, R. A., and Poljak, R. J. (1994). Structural and physicochemical analysis of the reaction between the anti-lysozyme antibody D1.3 and the anti-idiotopic antibodies E225 and E5.2. *J. Mol. Recognit.* 7, 57–62. doi:10.1002/jmr.300070108.

Tickenbrock, L., Kössmeier, K., Rehmann, H., Herrmann, C., and Müller, O. (2003). Differences between the interaction of beta-catenin with non-phosphorylated and single-mimicked phosphorylated 20-amino acid residue repeats of the APC protein. *J. Mol. Biol.* 327, 359–367.

Tong, K. I., Katoh, Y., Kusunoki, H., Itoh, K., Tanaka, T., and Yamamoto, M. (2006). Keap1 Recruits Neh2 through Binding to ETGE and DLG Motifs: Characterization of the Two-Site Molecular Recognition Model. *Molecular and Cellular Biology* 26, 2887–2900. doi:10.1128/MCB.26.8.2887-2900.2006.

Tran, B. N., Chen, L., Liu, Y., Wu, J., Velázquez-Campoy, A., Sivaraman, J., and Hew, C. L. (2011). Novel histone H3 binding protein ORF158L from the Singapore grouper iridovirus. *J. Virol.* 85, 9159–9166. doi:10.1128/JVI.02219-10.

Tsumoto, K., Ueda, Y., Maenaka, K., Watanabe, K., Ogasahara, K., Yutani, K., and Kumagai, I. (1994). Contribution to antibody-antigen interaction of structurally perturbed antigenic residues upon antibody binding. *J. Biol. Chem.* 269, 28777–28782.

Van Petegem, F., Duderstadt, K. E., Clark, K. A., Wang, M., and Minor, D. L. (2008). Alanine-scanning mutagenesis defines a conserved energetic hotspot in the CaValpha1 AID-CaVbeta interaction site that is critical for channel modulation. *Structure* 16, 280–294. doi:10.1016/j.str.2007.11.010.

Varadarajan, R., Connelly, P. R., Sturtevant, J. M., and Richards, F. M. (1992). Heat capacity changes for protein-peptide interactions in the ribonuclease S system. *Biochemistry* 31, 1421–1426.

Volkov, A. N., Bashir, Q., Worrall, J. A. R., and Ubbink, M. (2009). Binding hot spot in the weak protein complex of physiological redox partners yeast cytochrome C and cytochrome C peroxidase. *J. Mol. Biol.* 385, 1003–1013. doi:10.1016/j.jmb.2008.10.091.

Wang, H., Hota, P. K., Tong, Y., Li, B., Shen, L., Nedyalkova, L., Borthakur, S., Kim, S., Tempel, W., Buck, M., et al. (2011). Structural basis of Rnd1 binding to plexin Rho GTPase binding domains (RBDs). *J. Biol. Chem.* 286, 26093–26106. doi:10.1074/jbc.M110.197053.

Wang, J., Zhang, Z., Palzkill, T., and Chow, D.-C. (2007). Thermodynamic investigation of the role of contact residues of beta-lactamase-inhibitory protein for binding to TEM-1 beta-lactamase. *J. Biol. Chem.* 282, 17676–17684.

doi:10.1074/jbc.M611548200.

Weber, P. C., Pantoliano, M. W., and Thompson, L. D. (1992). Crystal structure and ligand-binding studies of a screened peptide complexed with streptavidin. *Biochemistry* 31, 9350–9354.

Wolfe, L. S., Stanley, B. J., Liu, C., Eliason, W. K., and Xiong, Y. (2010). Dissection of the HIV Vif interaction with human E3 ubiquitin ligase. *J. Virol.* 84, 7135–7139. doi:10.1128/JVI.00031-10.

Wu, J., Li, J., Li, G., Long, D. G., and Weis, R. M. (1996). The receptor binding site for the methyltransferase of bacterial chemotaxis is distinct from the sites of methylation. *Biochemistry* 35, 4984–4993. doi:10.1021/bi9530189.

Xu, Y., Oruganti, S. V., Gopalan, V., and Foster, M. P. (2012). Thermodynamics of coupled folding in the interaction of archaeal RNase P proteins RPP21 and RPP29. *Biochemistry* 51, 926–935. doi:10.1021/bi201674d.

Zheleva, D. I., Zhelev, N. Z., Fischer, P. M., Duff, S. V., Warbrick, E., Blake, D. G., and Lane, D. P. (2000). A quantitative study of the in vitro binding of the C-terminal domain of p21 to PCNA: affinity, stoichiometry, and thermodynamics. *Biochemistry* 39, 7388–7397. doi:10.1021/bi992498r.
